# Supplementary material for: Wnt signaling controls pro-regenerative Collagen XII in functional spinal cord regeneration in zebrafish
Source: Nat Commun. 2017 Jul 25;8:126. doi: 10.1038/s41467-017-00143-0 (PMC5526933; doi:10.1038/s41467-017-00143-0)
Supplement: Supplementary file 1 — Supplementary Information [file 41467_2017_143_MOESM1_ESM.pdf]

File Name: Peer Review File
Description:

File Name: Supplementary Information
Description: Supplementary Figures, Supplementary Tables, Supplementary Notes, Supplementary References.

File Name: Supplementary Movie 1
Description: < Axonal growth cones navigate independently of glial processes - example 1 > Representative examples of time-lapse video (n= 3) showing that axonal growth cones (labelled by Xla.Tubb:DsRED; red) extend into the lesion site independently of glia (labelled by her4.3:GFP; green). A dorsal view is shown. Rostral is left. Recording time is indicated in the movie as time post-lesion.

File Name: Supplementary Movie 2
Description: < Axonal growth cones navigate independently of glial processes - example 2 > Representative examples of time-lapse video (n= 6) showing that axonal growth cones (labelled by Xla.Tubb:DsRED; red) extend into the lesion site independently of glia (labelled by gfap:GFP; green). A lateral view is shown. Dorsal is up, rostral is left. Recording time is indicated in the movie as time post-lesion.

File Name: Supplementary Movie 3
Description: < Axon-Collagen I interaction > 3-D view showing regenerating axons (anti-acetylated Tubulin+ ; arrows), closely associated with Collagen I immunoreactivity in a lesion site (refers to Fig. 1g). A lateral view is shown. Dorsal is up, rostral is left.

File Name: Supplementary Movie 4
Description: < Axon-Fibronectin interaction > 3-D view showing regenerating axons (anti-acetylated Tubulin+ ; arrows), closely associated with Fibronectin immunoreactivity in a lesion site (refers to Fig. 1g). A lateral view is shown. Dorsal is up, rostral is left.

File Name: Supplementary Movie 5
Description: < Axon-Collagen XII interaction > 3-D view showing regenerating axons (anti-acetylated Tubulin+ ; arrows), closely associated with Collagen XII immunoreactivity in a lesion site (refers to Fig. 4i). A lateral view is shown. Dorsal is up, rostral is left.

File Name: Supplementary Data 1
Description: This file contains information on the source of in situ hybridization probe templates or primers used to generate in situ hybridization probe templates.

Supplementary Figures

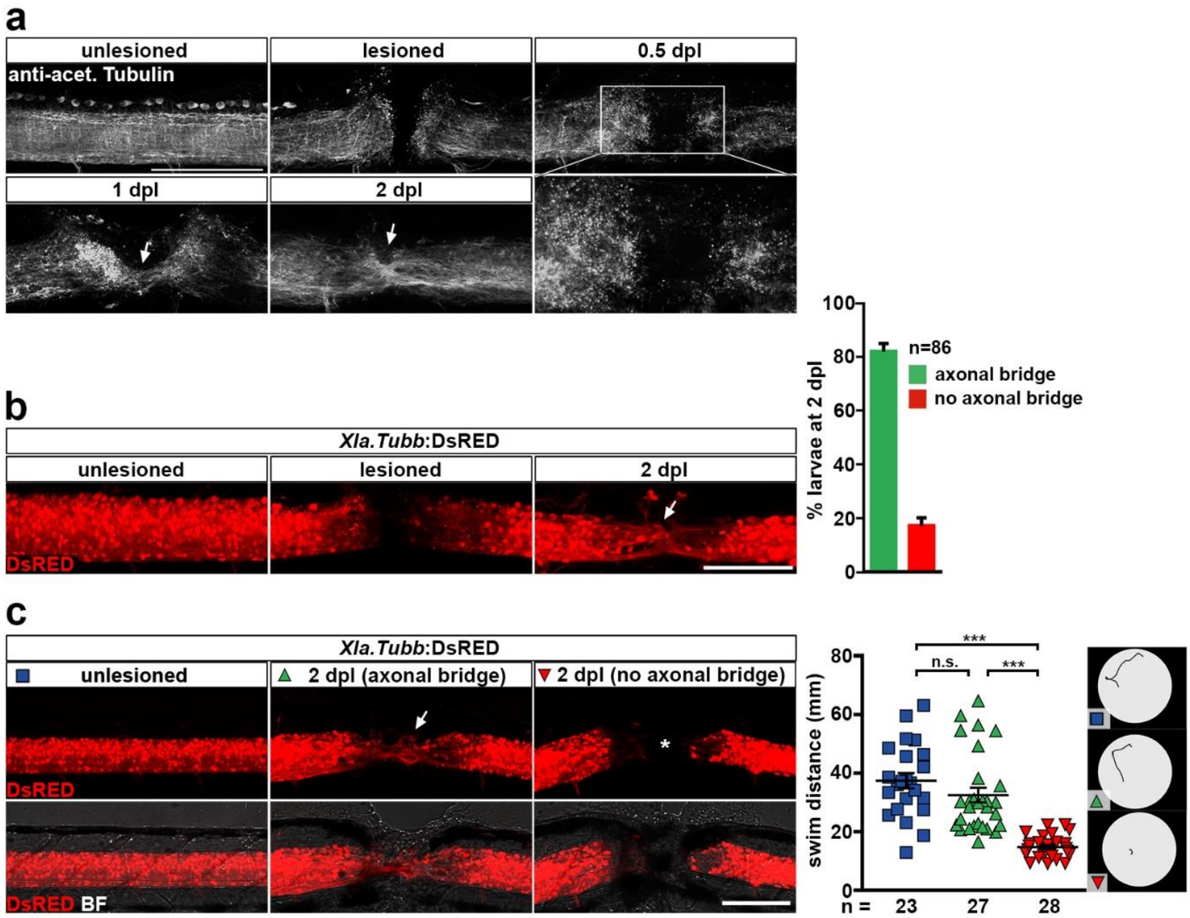

Supplementary Figure 1 | Functional spinal cord regeneration correlates with axon regrowth in zebrafish larvae.

(a) Time-course of axon regrowth (anti-acetylated Tubulin^{+}) after spinal cord transection. Note that at 0.5 dpl (12 hpl) little axon regrowth is evident and mainly axonal debris is found in the lesion site (inset). At 1 dpl axons begin to bridge the lesion, which is has substantially progressed at 2 dpl (arrows).

(b) Repetitive imaging of *Xla.Tubb*:DsRED transgenic larvae shows axonal bridge formation across the lesion site (arrow) in >80% of all animals analysed. Shown is the same animal before lesion, immediately after lesion and at 2 dpl.

(c) Functional recovery, as measured by the swim distance after touch, correlates with axon regrowth across the lesion site (axonal bridge formation) in larval zebrafish. Animals with an axonal bridge at 2 dpl (arrow) cover similar swim distance as unlesioned age-matched control animals. In animals in which no axonal reconnection is established by 2 dpl (asterisk), recovery of swim distance is impaired (one-way ANOVA with Dunn's multiple comparison test: ***$P<0.001$, n.s. indicates not significant). Example swim tracks are shown.

(a-c) Views are lateral (dorsal is up, rostral is left). BF: brightfield. Scale bars: 200 µm (c) and 100 µm (a-b). Error bars indicate s.e.m.

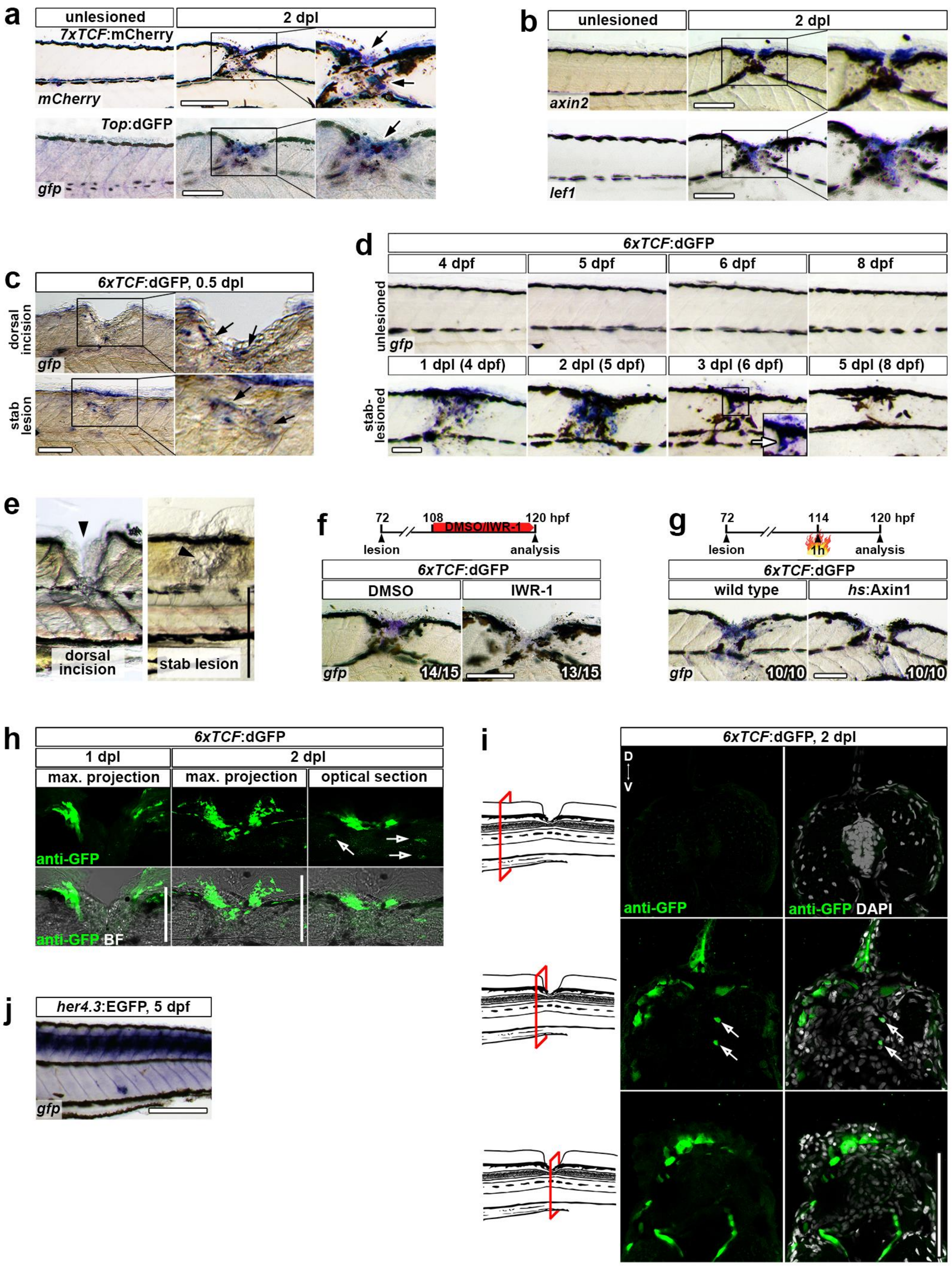

Supplementary Figure 2 | Wnt/β-catenin pathway is mainly active in the spinal lesion site.

(a) Expression of the transgenic Wnt/β-catenin pathway reporter *7xTCF*:mCherry and *Top*:dGFP is upregulated after lesion.

(b) Expression of direct Wnt target genes *axin2* and *lef1* is upregulated after lesion.

(c) *6xTCF*:dGFP Wnt reporter activity in the spinal lesion site is detectable 12 hours (0.5 dpl) after a stab lesion or dorsal incision lesion (arrows). Note that larvae were treated with PTU to suppress pigmentation.

(d) Detection of *gfp* mRNA in the *6xTCF*:dGFP transgenic reporter line shows transient activity of the Wnt/β-catenin pathway in the lesion site during regeneration, analysed in stab-lesioned animals.

(e) Representative images of a dorsal incision lesion or less invasive stab lesion taken immediately after lesion. Note that stab lesions do not compromise the dorsal edge of the animal, resulting in reduced injury size as compared to dorsal incision lesions. Arrowhead points to the lesion site.

(f) *6xTCF*:dGFP Wnt reporter activity in the lesion site is strongly reduced upon IWR-1 treatment for 12 hours, indicating specificity of the transgenic Wnt reporter.

(g) *6xTCF*:dGFP Wnt reporter activity in the lesion site is strongly reduced upon heat shock-induced *axin1* overexpression for 6 hours in *6xTCF*:dGFP;*hs*:Axin1 double transgenic animals, indicating specificity of the transgenic Wnt reporter.

(h) GFP protein is largely confined to the lesion site in *6xTCF*:dGFP transgenic animals at 1 dpl and 2 dpl. A few additional cells are labelled within the presumptive spinal cord region in the periphery of the lesion site (empty arrows). Maximum intensity projections and a single optical section through the center of a whole mount larvae are shown.

(i) Anti-GFP immunohistochemistry on sections of *6xTCF*:dGFP transgenic animals confirms labelling in whole mount preparations. *6xTCF*:dGFP Wnt reporter activity at 2 dpl is largely confined to the lesion site. In the peripheral lesion area limited Wnt reporter activity is also detected in the spinal cord (empty arrows).

(j) Detection of *gfp* mRNA by in situ hybridization in 5 day-old *her4.3*:EGFP transgenic zebrafish, labelling ependymoradial glia cells, reveals efficient probe penetration in whole mount preparations.

(a-j) Views are lateral (a-h, j; dorsal is up, rostral is left) or transversal (i; dorsal is up). BF: brightfield. Scale bars: whole mounts, 200 µm (j,e) and 100 µm (a-d, f-h); sections, 100 µm.

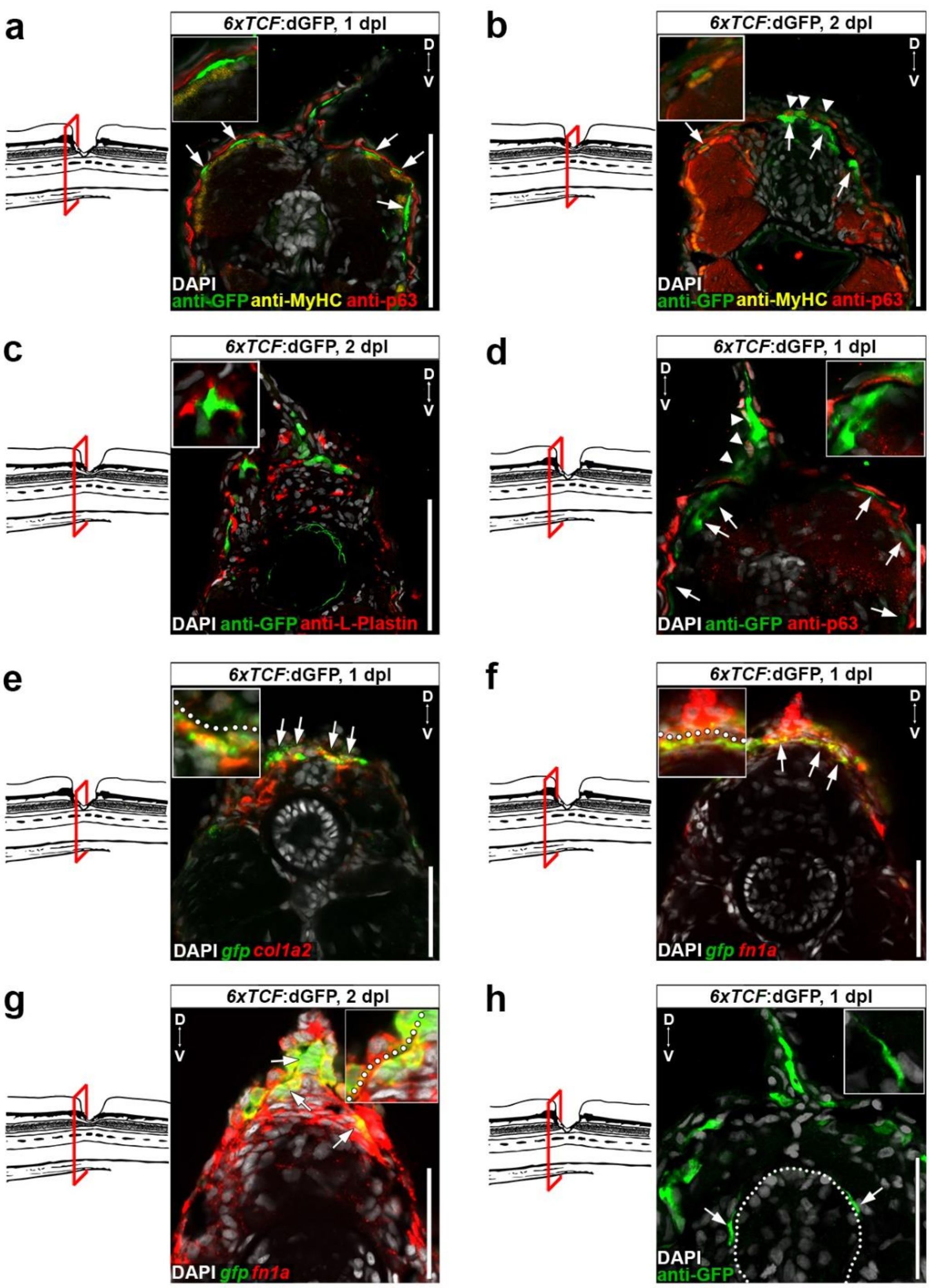

Supplementary Figure 3 | Wnt/β-catenin pathway is upregulated in basal keratinocytes and fibroblast-like cells but not muscle or immune cells after a lesion.

(a-b) Subepidermal *6xTCF*:dGFP^{+}/p63^{-} cells (arrows) do not co-label with MyHC antibodies, a marker for slow twitch muscle cells. Arrowheads indicate *6xTCF*:dGFP^{+}/p63^{+} basal keratinocytes. Note that close to the disorganized lesion center some subepidermal *6xTCF*:dGFP^{+}/p63^{-} cells are found sandwiched between p63^{+} basal keratinocytes and MyHC^{+} slow twitch muscle fibers (insets), which represents a typical dermal fibroblast location in zebrafish larvae.

(c) *6xTCF*:dGFP^{+} cells do not co-label with L-Plastin, a marker for innate immune cells.

(d) The *6xTCF*:dGFP Wnt reporter is active in p63^{+} basal keratinocytes (arrowheads) and in subepidermal p63^{-} cells (arrows) in the lesion site.

(e) Subepidermal cells (below dashed line) co-express *col1a2* (red) and *gfp m*RNA (green) in lesioned *6xTCF*:dGFP transgenic animals (arrow). Dashed line indicates junction between basal epithelium and subadjacent connective tissue.

(f-g) Subepidermal cells (below dashed line) co-express *fn1a* (red) and *gfp m*RNA (green) in lesioned *6xTCF*:dGFP transgenic animals (arrow). Dashed line indicates junction between basal epithelium and subadjacent connective tissue.

(h) Close to the disorganized lesion center, some *6xTCF*:dGFP$^+$ cells are found to cover the spinal cord (arrows), which represents a typical meningeal fibroblast location. Dashed line indicates spinal cord.

(a-h) Views are transversal (dorsal is up). Scale bars: 100 µm (a-c) and 50 µm (d-h).

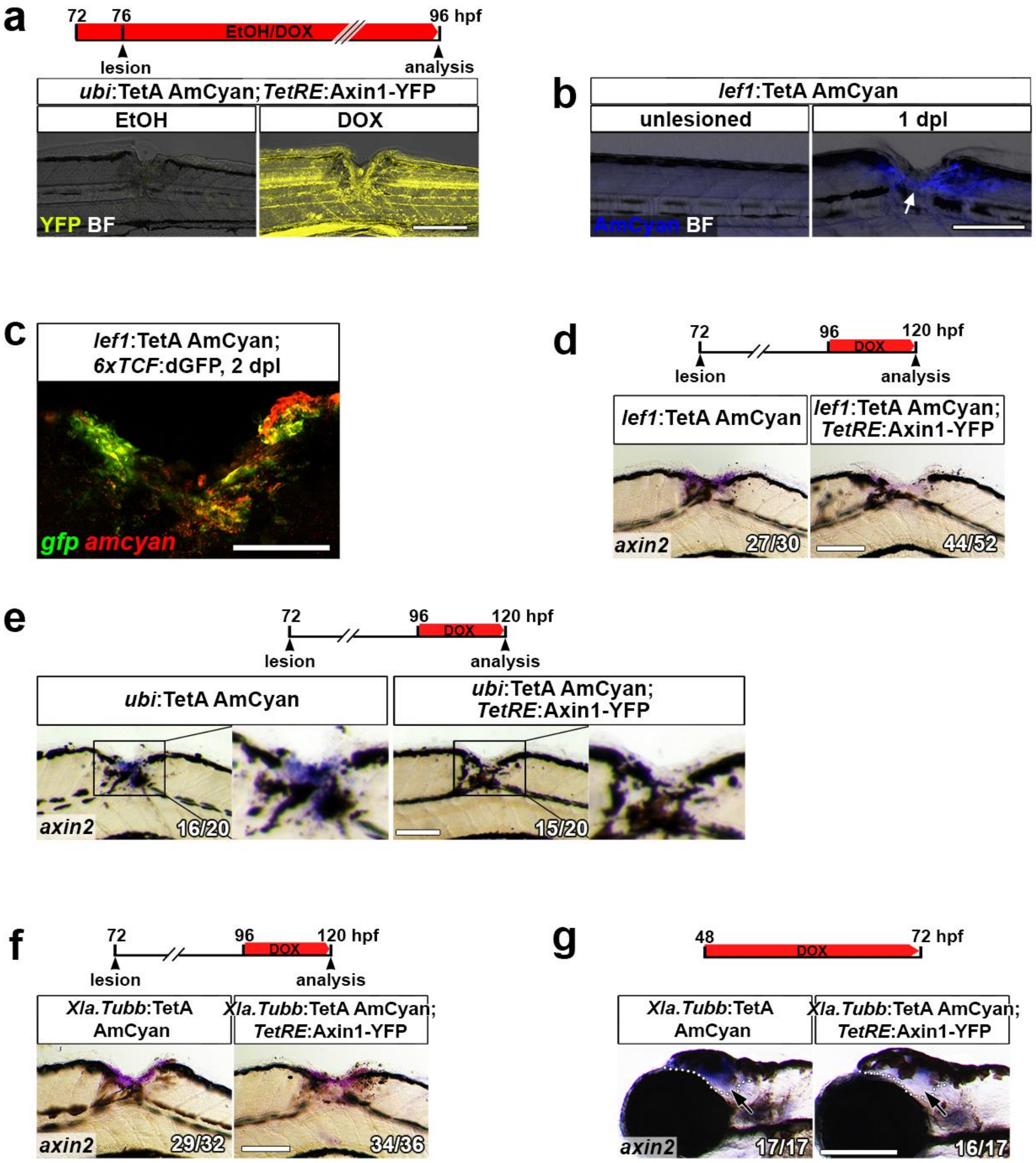

Supplementary Figure 4 | Utilizing the TetON system for cell type-specific Wnt/β-catenin pathway manipulation.

(a) The TetON system allows for inducible transgene expression. YFP fluorescence (TetResponder transgene) is robustly induced in DOX-treated but not EtOH-treated *ubi*:TetA AmCyan;*TetRE*:Axin1-YFP double transgenic animals.

(b) Consistent with Wnt/β-catenin pathway activation, AmCyan fluorescence is undetectable in unlesioned *lef1*:TetA AmCyan transgenic animals and upregulated after a lesion (arrow).

(c) *gfp* mRNA (green) and *amcyan* mRNA (red) are co-expressed in lesioned *6xTCF*:dGFP;*lef1*:TetA AmCyan double transgenic animals, indicating that *lef1* regulatory elements drive gene expression in Wnt-responding cells in the lesion site.

(d) DOX treatment of *lef1*:TetA AmCyan;*TetRE*:Axin1-YFP double transgenic animals (but not single transgenic control animals) interferes with *axin2* expression in non-neural lesion site cells.

(e) Ubiquitous *axin1* overexpression through DOX treatment of *ubi*:TetA AmCyan; *TetRE*:Axin1-YFP double transgenic animals, interferes with *axin2* expression in non-neural lesion site cells.

(f) *axin1* overexpression specifically in neurons through DOX treatment of *Xla.Tubb*:TetA AmCyan;*TetRE*:Axin1-YFP double transgenic animals does not reduce *axin2* expression in non-neuronal lesion site cells.

(g) DOX treatment of *Xla.Tubb*:TetA AmCyan;*TetRE*:Axin1-YFP double transgenic animals (but not single transgenic control animals) reduced *axin2* expression in a constitutively Wnt-responsive domain in the brain (arrows), indicating that the *Xla.Tubb*:TetA AmCyan TetActivator line drives functionally relevant *axin1* levels in neurons.

(a-g) Views are lateral (dorsal is up, rostral is left). BF: brightfield. Scale bars: whole mounts, 200 µm (g), 100 µm (a-b, d-f) and 25 µm (c).

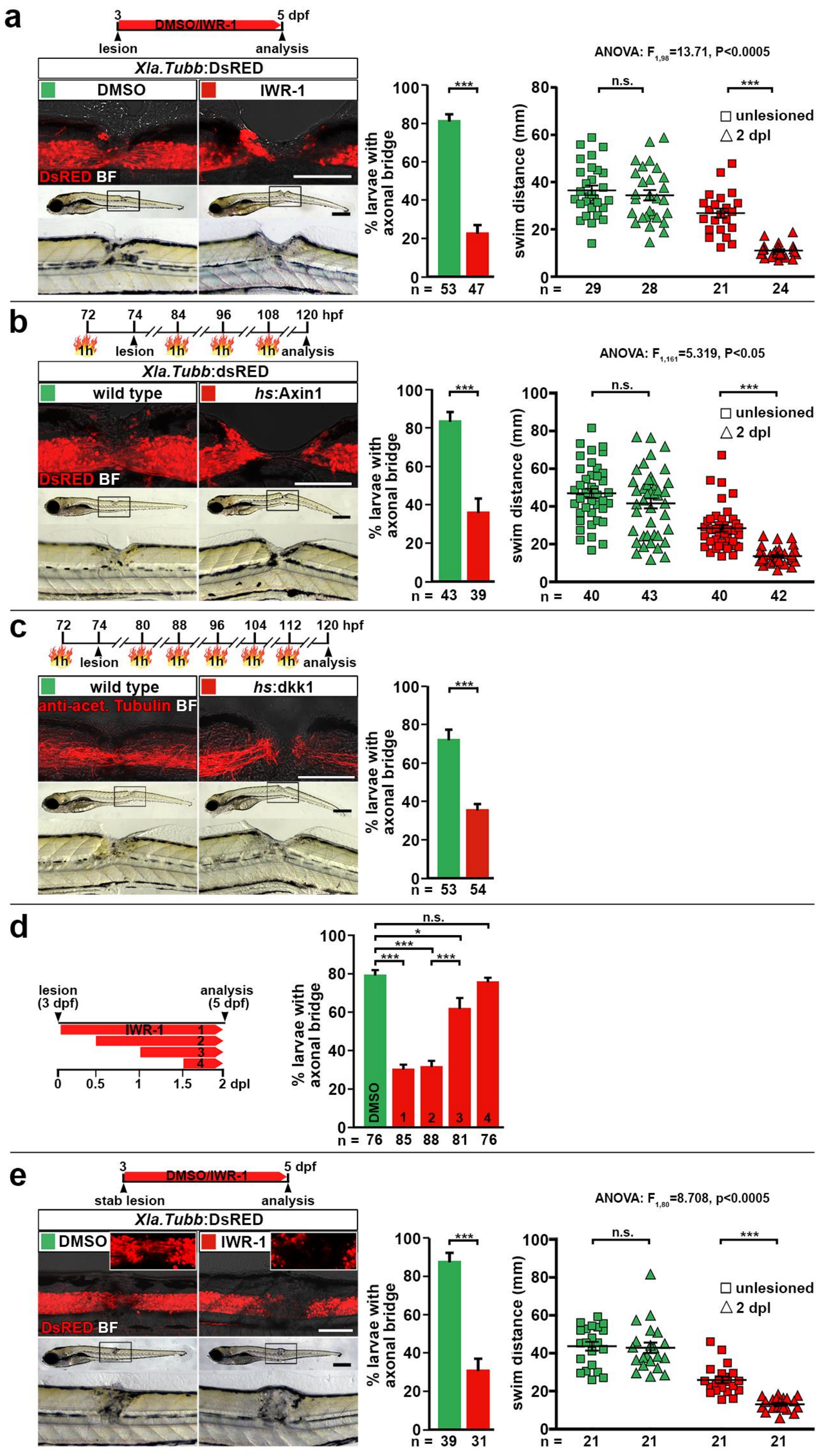
a
3
DMSO/IWR-1
5 dpf
lesion
analysis
Xla.Tubb:DsRED
DMSO
IWR-1
DsRED BF
% larvae with axonal bridge
n = 53 47
ANOVA: F1,98=13.71, P<0.0005
swim distance (mm)
unlesioned
2 dpl
n.s.
n = 29 28 21 24
b
72 74 84 96 108 120 hpf
lesion
analysis
Xla.Tubb:dsRED
wild type
hs:Axin1
n = 43 39
ANOVA: F1,161=5.319, P<0.05
n = 40 43 40 42
c
72 74 80 88 96 104 112 120 hpf
wild type
hs:dkk1
anti-acet. Tubulin BF
n = 53 54
d
lesion (3 dpf)
analysis (5 dpf)
IWR-1
0 0.5 1 1.5 2 dpl
DMSO
n = 76 85 88 81 76
e
stab lesion
ANOVA: F1,80=8.708, p<0.0005
n = 39 31
n = 21 21 21 21

Supplementary Figure 5 | Different lesion and manipulation paradigms support that Wnt/β-catenin signaling is required for axon regeneration and functional recovery after spinal cord lesion.

(a) Pharmacological interference (IWR-1) with Wnt/β-catenin signaling inhibits axon regeneration (Fischer's exact test: ***P<0.001) and functional recovery (two-way ANOVA: $F_{1,98}$=13.71, P<0.0005; t-test: ***P<0.001) in lesioned animals.

(b) Heat shock-induced ubiquitous overexpression of the Wnt/β-catenin pathway antagonist *axin1* inhibits axon regeneration (Fischer's exact test: ***P<0.001) and functional recovery (two-way ANOVA: $F_{1,161}$=5.319, P<0.05; t-test: ***P<0.001) in lesioned *hs*:Axin1 transgenic animals.

(c) Heat shock-induced ubiquitous overexpression of the Wnt/β-catenin pathway antagonist *dkk1* inhibits axon regeneration (Fischer's exact test: ***P<0.001) in lesioned *hs*:dkk1 transgenic animals.

(d) Pharmacological interference (IWR-1) with Wnt/β-catenin signaling starting at different time points post-lesion indicates that pathway activity is required between 0.5 dpl (12 hpl) and 1 dpl for axon regeneration (Fischer's exact test: *P<0.05, ***P<0.001, n.s. indicates not significant).

(e) Pharmacological (IWR-1) interference with Wnt/β-catenin signaling inhibits axon regeneration (Fischer's exact test: ***P<0.001) and functional recovery (two-way ANOVA: $F_{1,80}$=8.708, P<0.0005) in stab-lesioned animals.

(a-e) Views are lateral (dorsal is up, rostral is left. BF: brightfield. Scale bars: whole mounts, 200 µm and 100 µm. Error bars indicate s.e.m.

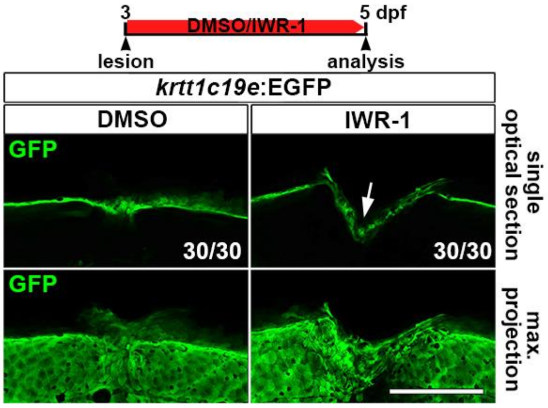

Supplementary Figure 6 | Wnt/β-catenin signaling is not required for reepithelialization of the lesion site.

Pharmacological (IWR-1) interference with Wnt/β-catenin signaling does not prevent re-epithelialization of the lesion site by basal keratinocytes (arrow), visualized in *krtt1c19e*:EGFP transgenic animals. Maximum intensity projections and single optical sections though the center of a whole mount larvae are shown. Views are lateral (dorsal is up, rostral is left). Scale bar: 200 µm.

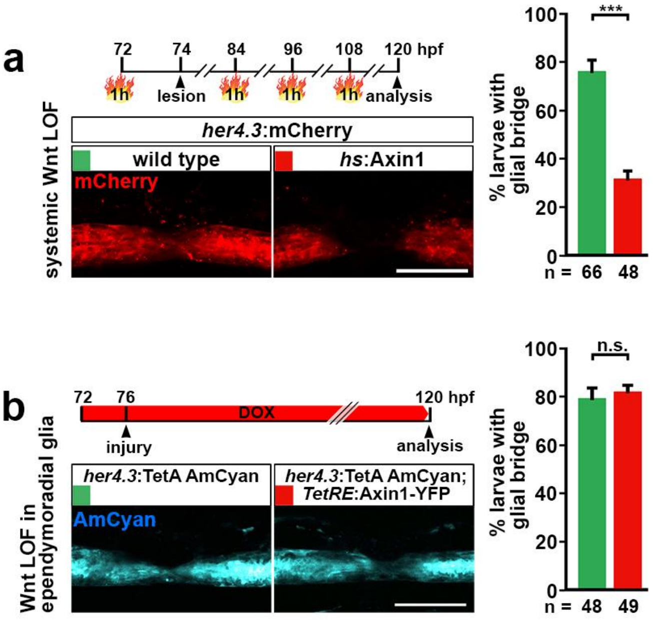

Supplementary Figure 7 | Non-cell autonomous Wnt/β-catenin signaling is required for glial bridging.

(a) Heat shock-induced ubiquitous overexpression of the Wnt/β-catenin pathway antagonist *axin1* inhibits glia growth (visualized in *her4.3*:mCherry transgenic animals) across the lesion site (Fischer's exact test: ****P*<0.001) in *hs*:Axin1 transgenic animals.

(b) *axin1* overexpression specifically in ependymoradial glial cells through DOX treatment of *her4.3*:TetA AmCyan;*TetRE*:Axin1-YFP double transgenic fish does not interfere with glial growth across the lesion site (Fischer's exact test: n.s. indicates not significant).

(a-b) Views are lateral (dorsal is up, rostral is left). Scale bar: 100 µm. Error bars indicate s.e.m.

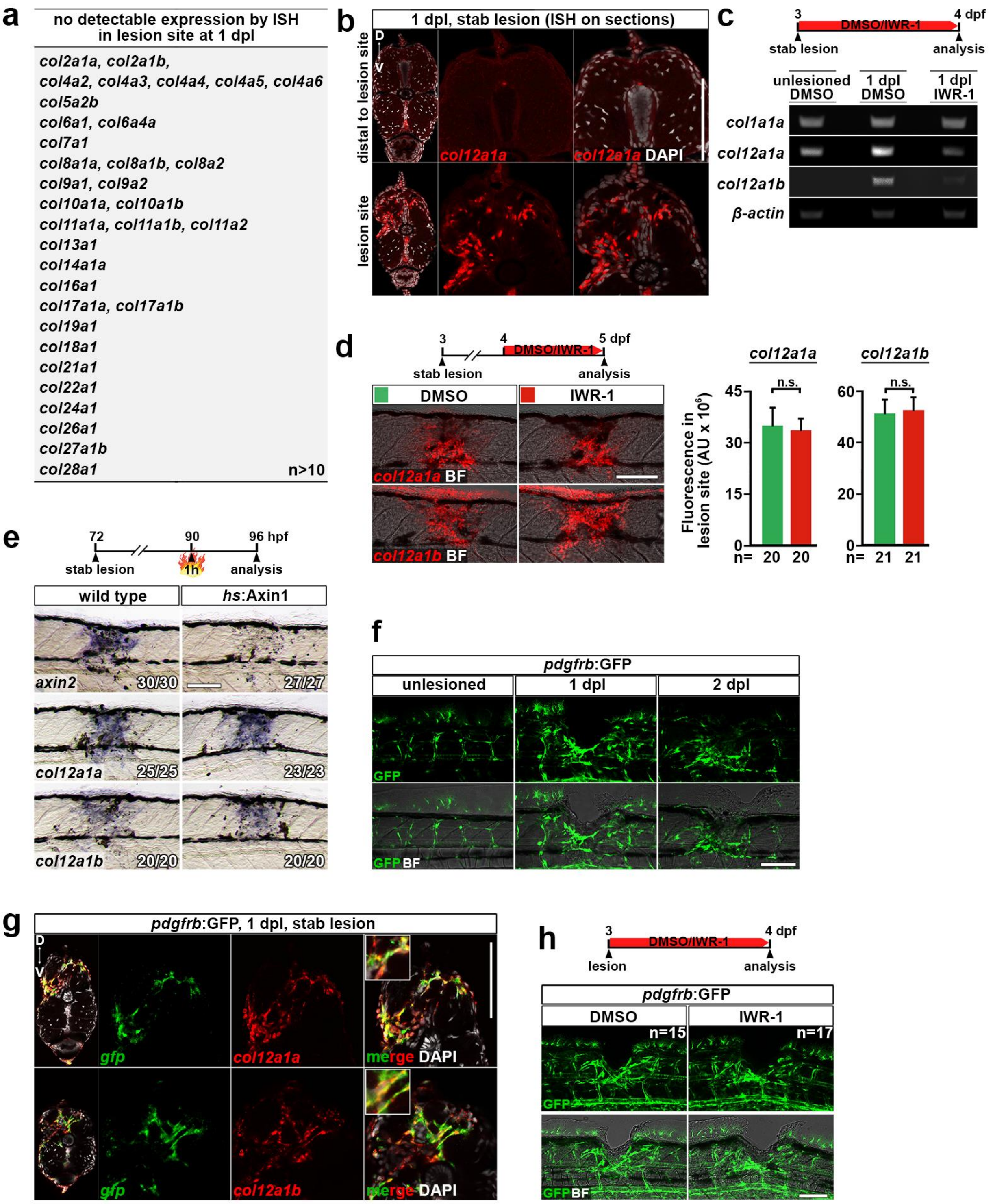

Supplementary Figure 8 | Wnt/β-catenin signaling selectively controls *col12a1a/b* transcription in a spinal lesion site during the initial phase of axon regeneration.

(a) List of genes encoding collagen-chains that were not detectably expressed in the lesion site of untreated or Wnt/β-catenin pathway-inhibited (IWR-1) animals at 1 dpl by in situ hybridization.

(b) In situ hybridization (ISH) on section confirms that *col12a1a* expression is confined to the lesion site.

(c) RT-PCR of trunk tissue surrounding the lesion site confirms that lesion-induced upregulation of *col12a1a/b* expression depends on Wnt/β-catenin signaling.

(d) Inhibition of Wnt/β-catenin signaling (IWR-1) between 1-2 dpl does not affect expression of *col12a1a/b* in the lesion site, as determined by quantification of fluorescence in situ hybridization signal (t-test: n.s. indicates not significant).

(e) Expression of *axin2*, a direct Wnt/β-catenin target, but not of *col12a1a* or *col12a1b* is strongly reduced in the lesion site within 6 hours of pathway inhibition by heat shock induced overexpression of *axin1* in *hs*:Axin1 transgenic animals. This suggests indirect transcriptional control of *col12a1a/b* genes by the Wnt/β-catenin pathway.

(f) *pdgfrb*:GFP-expressing cells accumulate in the lesion site after lesion.

(g) The majority of *col12a1a/b*-expressing cells (red) co-express *gfp* mRNA (green) in lesioned *pdgfrb*:GFP transgenic animals (arrow), a marker for pericytes and reactive fibroblasts.

(h) Interference with Wnt/β-catenin signaling (IWR-1) does not overtly inhibit appearance of *pdgfrb*:GFP$^+$ cells.

(a-h) Views are lateral (d-f, h; dorsal is up, rostral is left) or transversal (b, g; dorsal is up). BF: brightfield. Scale bars: whole mounts, 100 µm; sections, 100 µm. Error bars indicate s.e.m.

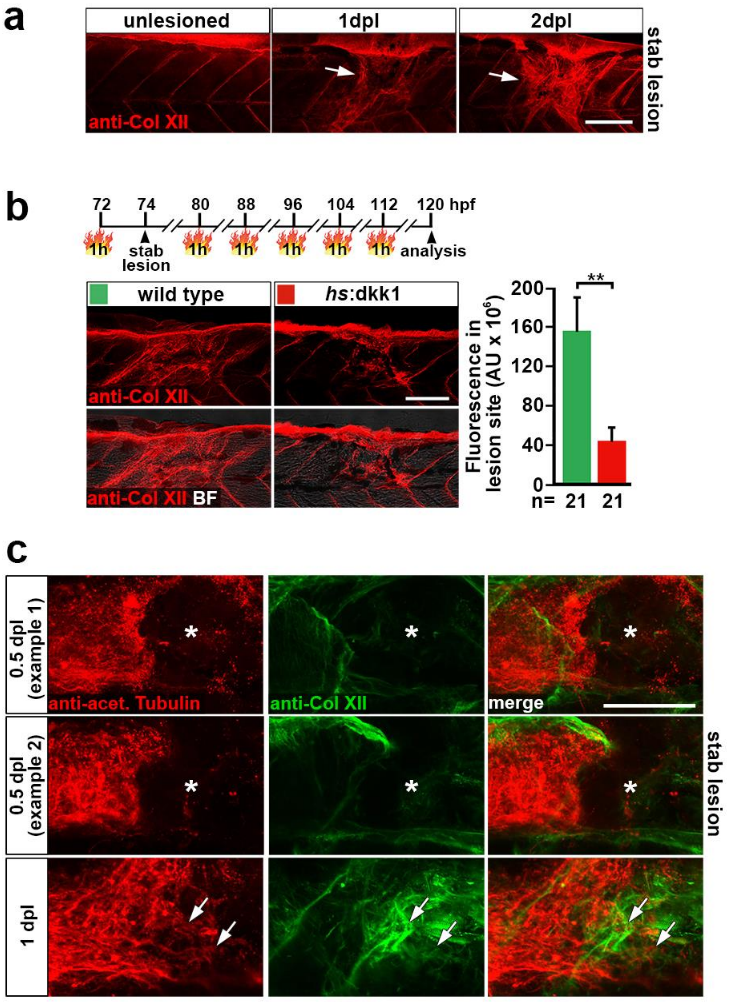

Supplementary Figure 9 | Wnt/β-catenin signaling controls Col XII matrix deposition in a spinal lesion site through which regenerating axons grow.

(a) Anti-Col XII immunoreactivity is increased in the lesion site (arrows) at 1 dpl and 2 dpl compared to adjacent unlesioned tissue and to unlesioned animals at the same trunk position.

(b) Inhibition of Wnt/β-catenin signaling via heat shock-induced systemic overexpression of the pathway antagonist *dkk1* interferes with Col XII deposition in the lesion site, as determined by quantification of lesion site immunoreactivity (t-test: $^{**}P<0.01$).

(c) Double immunolabelling of axons (anti-acetylated Tubulin^{+}) and Col XII shows little to no Col XII deposition in the lesion site at 0.5 dpl (12 hpl) and axon are yet to enter the lesion site (asterisk). At 1 dpl, anti-Col XII immunoreactivity is markedly increased in the lesion site and axon have entered the Col XII-rich lesion site (arrow). Confocal depth was limited to spinal cord. Note, that the 1 dpl dataset is the same as presented in Fig. 4f.

(a-c) Views are lateral (dorsal is up, rostral is left). BF: brightfield. Scale bars: whole mounts, 100 µm (a-b) and 50 µm (c). Error bars indicate s.e.m.

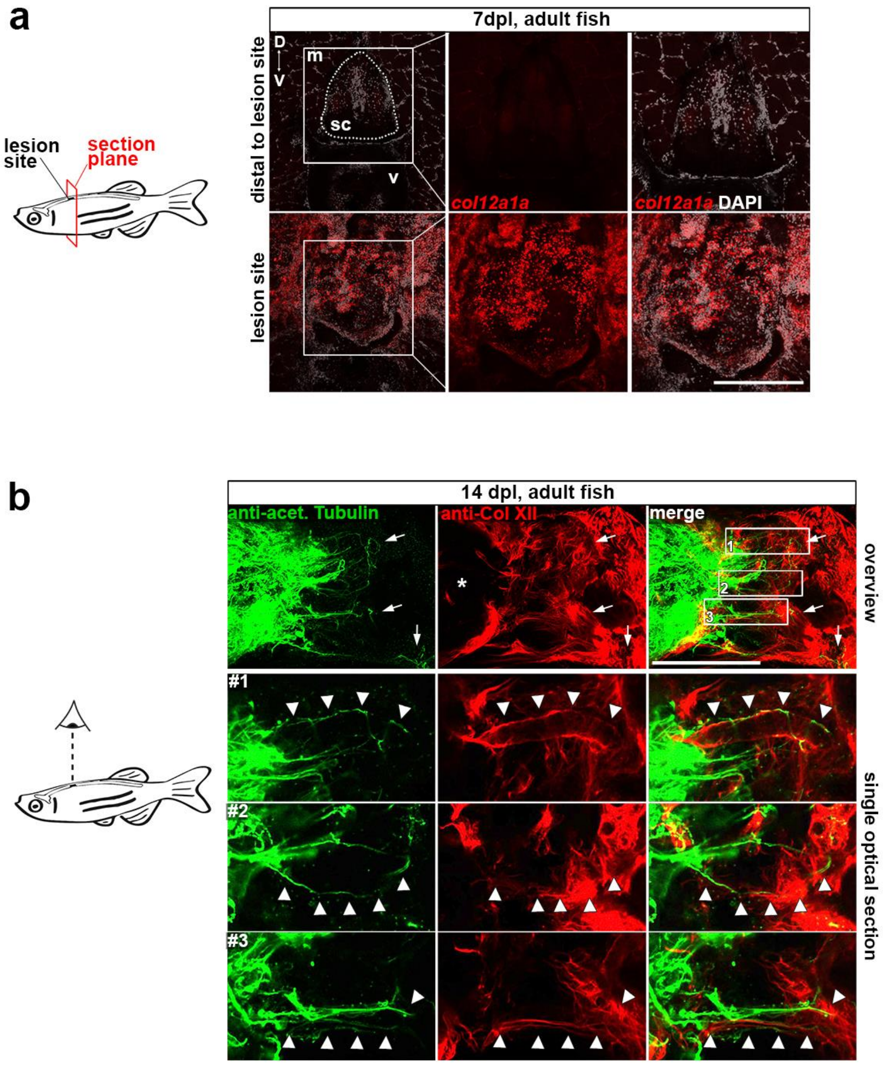

Supplementary Figure 10 | Col XII matrix deposition in a spinal lesion site is conserved across developmental stages in zebrafish.

(a) In adult zebrafish, *col12a1a* is strongly expressed in a spinal lesion site (bottom panel). Transcripts are undetectable in the intact spinal cord distal to the lesion site (top panel). Abbreviations: sc, spinal cord; m, muscle; v, vertebra.

(b) In adult zebrafish, regenerating axons (anti-acetylated Tubulin^{+}) navigate a Col XII-rich lesion environment (arrows). Note that Col XII immunoreactivity is undetectable in the intact portion of the spinal cord (asterisk). Shown is a maximum intensity projection (82 µm) and single optical sections with higher magnification showing close association between axons and Col XII. Note that the trajectory of many axonal fascicles appear to follow longitudinal fibres of Col XII immunoreactive ECM material in the lesion site (arrowheads).

(a-b) Views are transversal (a; dorsal is up) or dorsal (b; rostral is left). Scale bars: 250 µm (a), 200 µm (b).

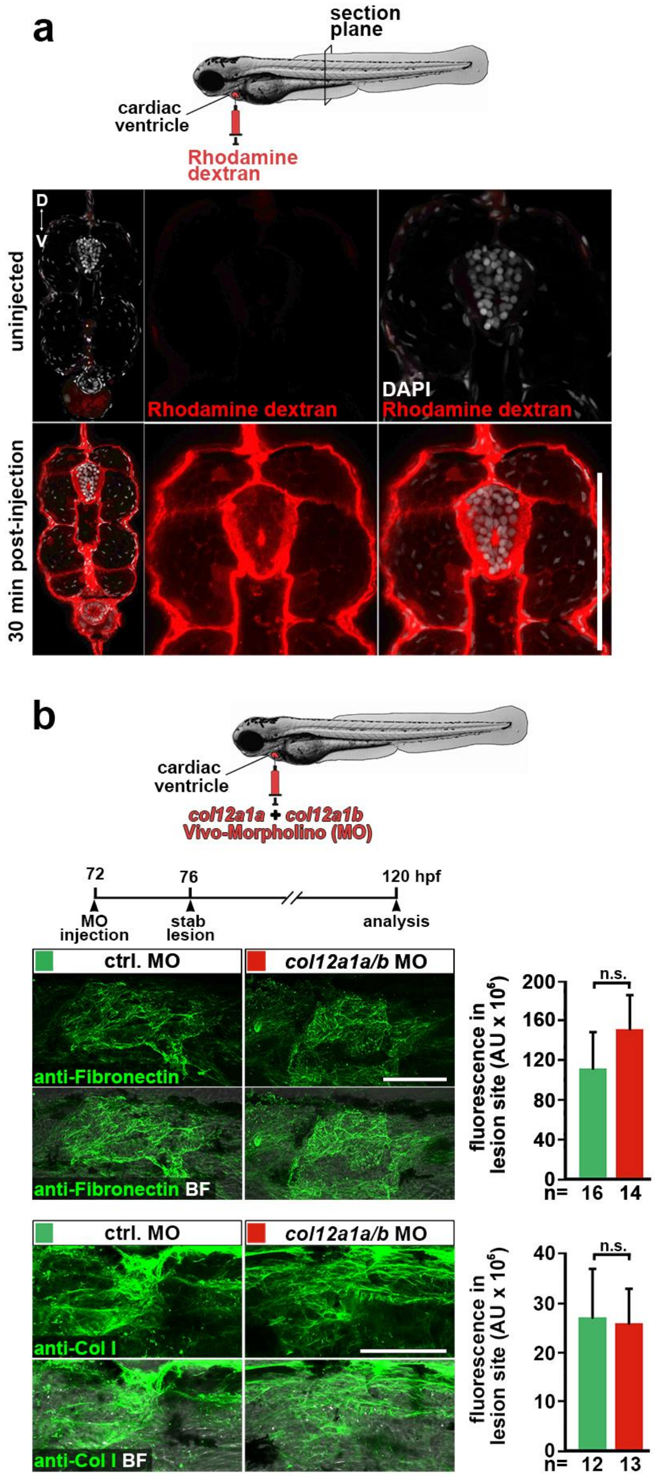

Supplementary Figure 11 | Systemic administration of *col12a1a/b* Vivo-Morpholinos does not interfere with Fibronectin or Collagen I deposition in the lesion site.

(a) Rhodamine dextran injected into the cardiac ventricle of 3 dpf zebrafish is efficiently distributed in the extracellular space throughout the animal within 30 min after injection.

(b) Injection of *col12a1a/b* Vivo-Morpholinos (MO) does not affect anti-Fibronectin (top panel) or anti-Collagen I (Col I; bottom panel) immunoreactivity in the lesion site (t-test: n.s. indicates not significant).

(a-b) Views are transversal (a; dorsal is up) or lateral (b; dorsal is up, rostral is left). BF: brightfield. Scale bars: whole mounts, 100 µm; sections, 100 µm. Error bars indicate s.e.m.

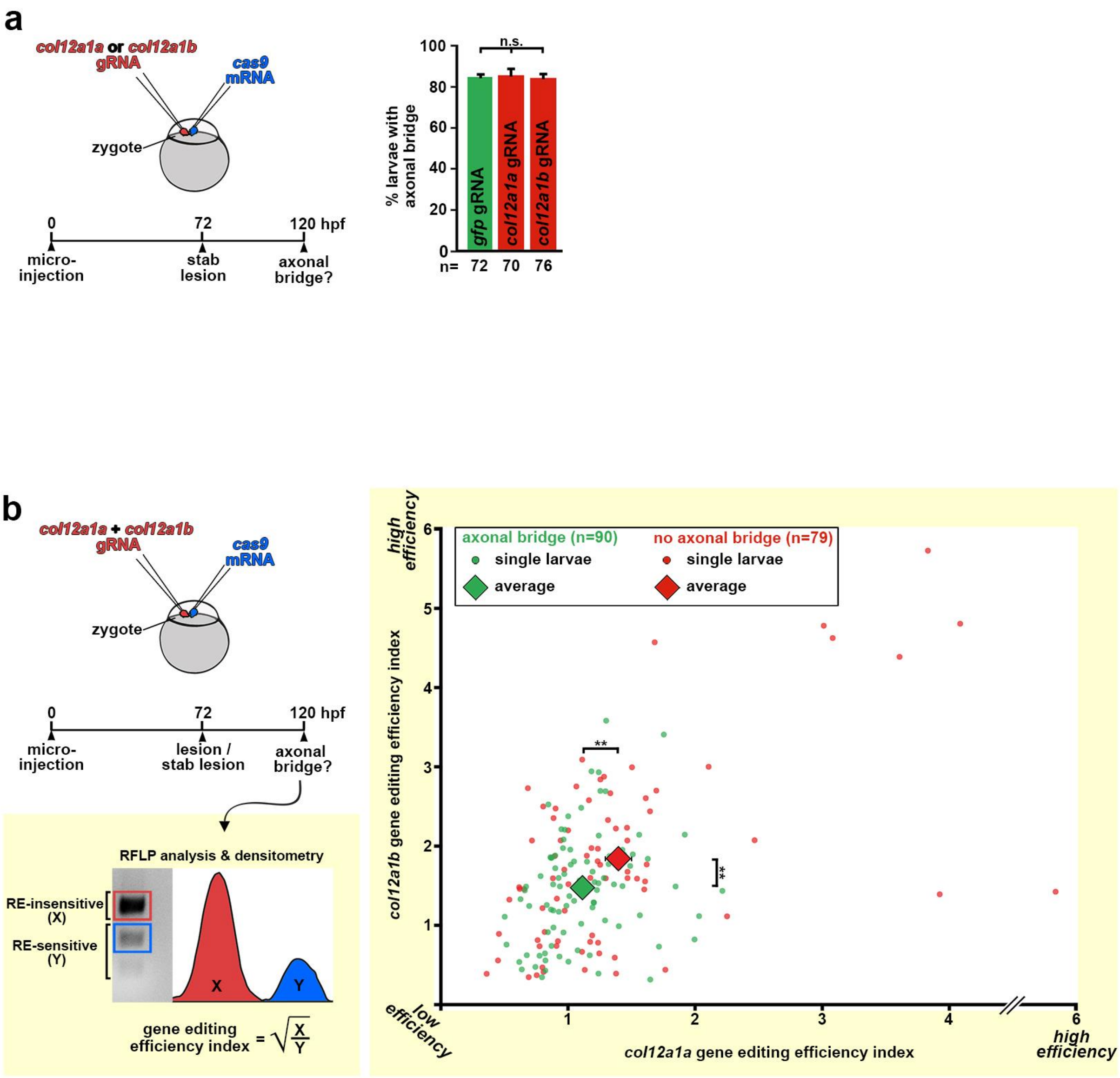

Supplementary Figure 12 | CRISPR/Cas9-mediated disruption of *col12a1a/b* leads to impaired axonal regeneration.

(a) Transient CRISPR/Cas9-mediated gene editing of either *col12a1a* or *col12a1b* does not affect axonal regeneration (Fischer's exact test: n.s. indicates not significant).

(b) Lack of axonal regeneration correlates with high gene editing efficiency of both *col12a1* paralogues in CRISPR -manipulated larvae. Single larvae were analysed for axonal bridging at 2 dpl followed by restriction fragment length polymorphism (RFLP) analysis and determination of the gene edition efficiency index. Larvae without axonal bridge at 2 dpl show on average higher gene editing efficiency of *col12a1a* and *col12a1b* than larvae with axonal bridge (t-test: $**P<0.01$). Abbreviation: RE, restriction endonuclease. Error bars indicate s.e.m.

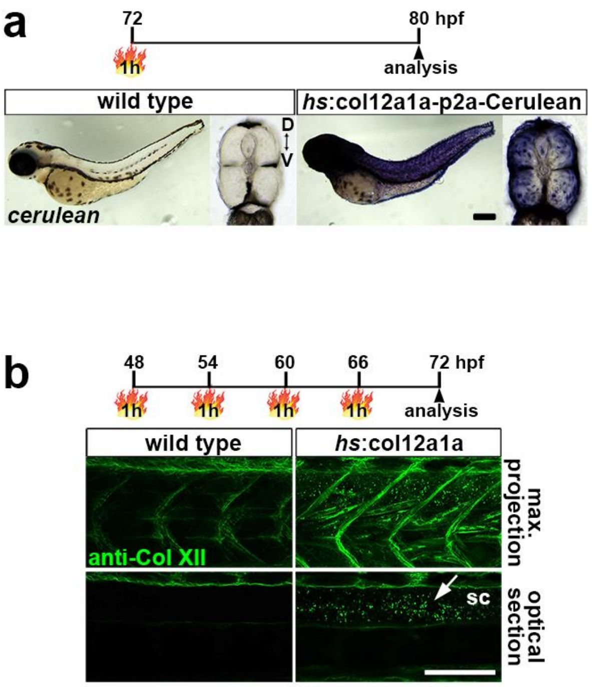

Supplementary Figure 13 | Using *hs*:col12a1a-p2a-Cerulean transgenic fish for inducible *col12a1a* overexpression

(a) *cerulean* mRNA is robustly induced after a single heat shock in *hs*:col12a1a-p2a-Cerulean transgenic larvae.

(b) *col12a1a* overexpression increases anti-Col XII immunoreactivity in developmental Col XII domains and in ectopic sites, including the spinal cord (arrow). Shown is a maximum intensity projection and a single optical section at the level of the spinal cord. Abbreviations: sc, spinal cord.

(a-b) Views are lateral (dorsal is up, rostral is left) or transversal (section view in a; dorsal is up). BF: brightfield. Scale bars: 100 µm.

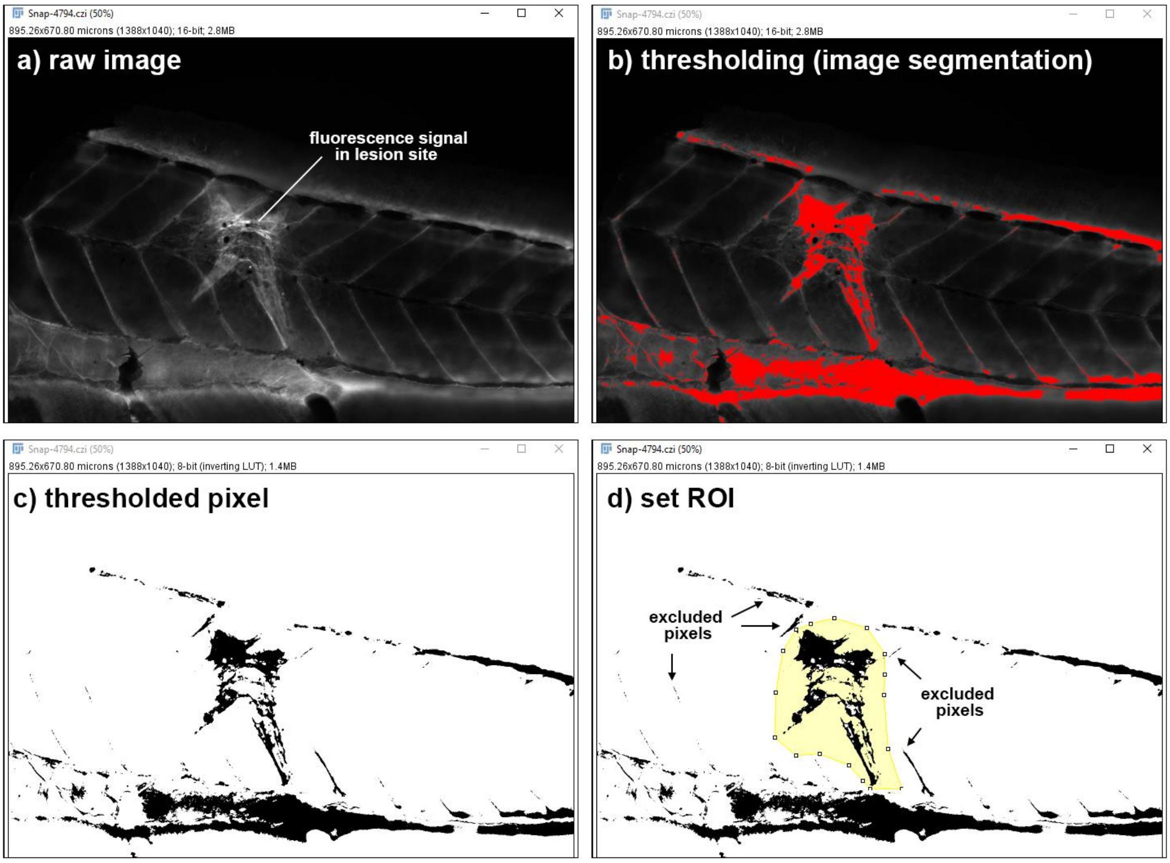

Supplementary Figure 14 | Workflow for the quantitative analysis of immunohistochemistry or fluorescence in situ hybridization signals in a lesion site.

A minimum intensity threshold is applied to the raw image **(a-b)**, limiting pixels to only those of equal or higher intensity **(c)**. After thresholding, a ROI is defined manually in which the thresholded pixel area is measured **(d)**. The analysis is done without knowledge of the experimental condition.

Supplementary Notes

Supplementary Note 1 | Supplemental information on transgenic zebrafish lines used.

Detection of Wnt/β-catenin pathway activity

***6xTCF/Lef-miniP*:2dGFP (abbreviated as *6xTCF*:dGFP)**

Reporter of β-catenin-dependent transcription. Destabilized EGFP under the control of a pGL4 minimal promoter plus 6 consensus Tcf/Lef binding sites. Previously shown to reliably report Wnt/β-catenin signaling during zebrafish development and adult zebrafish tail fin regeneration [1,2]. In this study lesion-induced *6xTCF*:dGFP reporter activity is specific for Wnt/β-catenin signaling because reporter activity is suppressed after overexpression of the pathway antagonist *axin1* for 6 hours or after 12 hours of treatment with the pharmacological pathway inhibitor IWR-1 (see Supplementary Fig. 2f-g).

***7xTCF-Xla.Siam*:nlsmCherry (abbreviated as *7xTCF*:mCherry)**

Reporter of β-catenin-dependent transcription. Monomeric mCherry fused to nuclear localization signal under the control of a *siamois* minimal promoter plus 7 consensus Tcf/Lef binding sites. Previously shown to reliably report Wnt/β-catenin signaling during zebrafish development and adult zebrafish tail fin regeneration [2,3].

***Top*:dGFP**

Reporter of β-catenin-dependent transcription. Destabilized GFP under the control of the TOPFLASH promoter containing 4 consensus Tcf/Lef binding sites. Previously shown to reliably report Wnt/β-catenin signaling during zebrafish development and caudal fin regeneration [2,4,5].

Systemic manipulation of Wnt/β-catenin pathway activity

***hsp70l*:dkk1-GFP (abbreviated as *hs*:dkk1)**

Zebrafish *dkk1b* (secreted antagonist of Wnt/β-catenin signaling) fused to EGFP at the C-terminus under the control of a heat shock-inducible promoter. Potently inhibits Wnt/β-catenin signaling when activated (heat shock) during zebrafish development or adult zebrafish tail fin regeneration [2,5]. Dkk1 specifically inhibits Wnt/β-catenin signalling through binding to Lrp5/6 Wnt co-receptors [6].

***hsp70l*:Mmu.Axin1-YFP (abbreviated as *hs*:Axin1)**

Mouse *axin1* (cytoplasmic antagonist of Wnt/β-catenin signaling) lacking the N-terminal RGS domain fused at the C-terminus to YFP under the control of a heat shock-inducible promoter. Potently inhibits Wnt/β-catenin signaling when activated (heat shock) during zebrafish development or adult zebrafish tail fin regeneration [2,7]. Axin1 inhibits Wnt/β-catenin signaling by facilitating degradation of the transcriptional co-activator β-catenin [6].

***hspl70l*:wnt8a-GFP (abbreviated as *hs*:wnt8)**

Zebrafish *wnt8* (Wnt ligand activating Wnt/β-catenin signaling) fused to GFP at the C-terminus under the control of a heat shock-inducible promoter. Potently activates Wnt/β-catenin signaling when activated (heat shock) during zebrafish development or adult zebrafish tail fin regeneration [5,8].

Cell type-specific manipulation of Wnt/β-catenin pathway activity (TetON system)

***her4.3*:irtTAM2(3F)-p2a-AmCyan (abbreviated as *her4.3*:TetA AmCyan)**

Astroglia-like cell-specific TetActivator line. Tetracycline-inducible transcriptional activator tagged with p2a and AmCyan under the control of regulatory sequences of the *her4.3* gene known to be selectively expressed in astroglia-like cells [9].

***lef1*:irtTAM2(3F)-p2a-AmCyan (abbreviated as *lef1*:TetA AmCyan)**

Tetracycline-inducible transcriptional activator tagged with p2a and AmCyan under the control of regulatory sequences of the direct Wnt/β-catenin target gene *lef1* [10,11]. This directs transgene expression to cells in which the Wnt pathway is active.

***ubiquitin*:irtTAM2(3F)-p2a-AmCyan (abbreviated as *ubi*:TetA AmCyan)**

TetActivator line for ubiquitous transgene expression. Tetracycline-inducible transcriptional activator tagged with p2a and AmCyan under the control of regulatory sequences of the *ubiquitin* gene [12].

***Xla.Tubb*:irtTAM2(3F)-p2a-AmCyan (*Xla.Tubb*:TetA AmCyan)**

Neuronal lineage-specific TetActivator line. Tetracycline-inducible transcriptional activator tagged with p2a and AmCyan under control of the Xenopus laevis neural-specific beta tubulin regulatory element (*Xla.Tubb2b*) [13].

***TetRE*:Mmu.Axin1-YFP (abbreviated as *TetRE*:Axin1-YFP)**

Mouse *axin1* (cytoplasmic antagonist of Wnt/β-catenin signaling) lacking the N-terminal RGS domain fused at the C-terminus to YFP under the control of a tetracycline response element. Potently inhibits Wnt/β-catenin signaling during zebrafish development or adult zebrafish tail fin regeneration when induced via a Tet Activator expressing transgene [2,14].

Inducible targeted cell ablation

***gfap*:Gal4ff;*UAS-E1b*:Eco.NfsB-mCherry (abbreviated as *gfap*:Gal4ff;*UAS*:NTR-mCherry)**

Zebrafish line for inducible ablation of astroglia-like cells [15]. Regulatory elements of the *gfap* gene driving bacterial Nitroreductase (NTR) via the Gal4/UAS system. NTR catalyses the reduction of the innocuous pro-drug Metronidazole (MTZ) to a cytotoxic product that induces cell death [16].

Fluorescent reporter lines, other

gfap:GFP

Astroglia-like cell reporter line. Cytoplasmic GFP under the control of *glial fibrillary acidic protein* (*gfap)* regulatory sequences. Labels astroglia-like cells in the zebrafish spinal cord [17].

her4.3:EGFP (previously known as _her4.1_)

Astroglia-like cell reporter line. Cytoplasmic GFP under the control of *her4* regulatory sequences. Labels astrocyte-like glial cells in the zebrafish spinal cord [9].

her4.3:Tet-GBD-p2A-mCherry (previously known as _her4.1_; abbreviated as _her4.3_:mCherry)

Astroglia-like cell-specific TetActivator line. Tetracycline-inducible transcriptional activator tagged with p2a and mCherry under the control of regulatory sequences of the *her4* gene. Used as astroglia-like cell reporter line only in this study.

hsp70l:col12a1a-p2a-Cerulean (abbreviated as _hs_:col12a1a)

Zebrafish full length *col12a1a* CDS tagged with p2a and Cerulean under the control of a heat shock-inducible promoter. Allows for systemic *col12a1a* overexpression when activated by heat shock.

krtt1c19e:EGFP

Basal keratinocyte reporter line. Cytoplasmic GFP under the control of regulatory sequences of the *krtt1c19e* gene. Labels p63+ basal keratinocytes in 3-5 day-old larvae [18].

pdgfrb:Gal4ff;_UAS_:GFP (abbreviated as _pdgfrb_:GFP)

Fluorescence reporter line labelling mural cells [19] (vascular smooth muscle cells, pericytes) and reactive fibroblasts [20]. Regulatory elements of the *pdgfrb* gene driving cycoplasmic GFP via the Gal4/UAS system.

Xla.Tubb:DsRED

Neuronal lineage reporter line. Cytoplasmic DsRED under the control of the Xenopus laevis neural-specific beta tubulin regulatory element (*Xla.Tubb2b*) [13].

Supplementary Tables

Supplementary Table 1 | Primer used for amplification of full *col12a1a* CDS.

fragment	size	forward primer	reverse primer
1	1765 bp	CGATGGCTTCCAGTTCGAGT	GCGACGTACAACCATCCTCA
2	2535 bp	GCTACCCAGGTTTTGCGTAGT	GTTTGACATGCGTCCTCAGC
3	4893 bp	CTTCACGAGGTGTGGACGAA	TGACGGAGGCATTACTTGGG

Supplementary Table 2 | Primer used for RT-PCR and q-RT-PCR.

gene	Ensembl ID	forward primer	reverse primer
col1a1a	ENSDART00000009393.7	CGATGGCTTCCAGTT CGAGT	GCGACGTACAACCATC CTCA
col12a1a	ENSDART00000154728.2	GCTACCCAGGTTTTG CGTAGT	GTTTGACATGCGTCCT CAGC
col12a1b	ENSDART00000025926.8	CTTCACGAGGTGTGG ACGAA	TGACGGAGGCATTACT TGGG
β-actin	ENSDART00000054987.6	GATCAAGATCATTGC TCCCCCTG	ATGCGCCATACAGAGC AGAA

Supplementary Table 3 | Primary antibodies used.

antibody name	raised in	source	catalog#	specificity reference
anti-Col I	rabbit	Abcam	Ab23730	[21]
anti-Col XII	rabbit	Florence Ruggiero, Université de Lyon		[22]
anti-Digoxigenin-AP, Fab fragments	sheep	Roche	11093274910	-

**antibody name**	**raised in**	**source**	**catalog#**	**specificity reference**
anti-Digoxigenin-POD, Fab fragments	sheep	Roche	11207733910	-
Anti-Fibronectin	rabbit	Sigma-Aldrich	F3648	[23]
anti-Fluorescein-AP, FAB fragments	sheep	Roche	11426338910	-
anti-Fluorescein-POD, FAB fragments	sheep	Roche	11426346910	-
anti-GFP	chicken	Abcam	ab13970	-
anti-Glial Fibrillary Acidic Protein	rabbit	Dako	Z0334	[24]
Anti-Laminin	Rabbit	Sigma-Aldrich	L9393	[25]
anti-L-Plastin	rabbit	Yi Feng, University of Edinburgh		[26]
anti-Myosin, heavy chain	mouse	DSHB	F59	[27]
anti-tp63	rabbit	Sigma-Aldrich	SAB2701838	this study
anti-Tubulin, acetylated (clone 6-11B-1)	mouse	Sigma-Aldrich	T6793	[24]

Supplementary References

1 Shimizu, N., Kawakami, K. & Ishitani, T. Visualization and exploration of Tcf/Lef function using a highly responsive Wnt/beta-catenin signaling-reporter transgenic zebrafish. *Dev Biol* **370**, 71-85, doi:10.1016/j.ydbio.2012.07.016 (2012).

2 Wehner, D. *et al.* Wnt/beta-catenin signaling defines organizing centers that orchestrate growth and differentiation of the regenerating zebrafish caudal fin. *Cell Rep* **6**, 467-481, doi:10.1016/j.celrep.2013.12.036 (2014).

3 Moro, E. *et al.* In vivo Wnt signaling tracing through a transgenic biosensor fish reveals novel activity domains. *Dev Biol* **366**, 327-340, doi:10.1016/j.ydbio.2012.03.023 (2012).

4 Dorsky, R. I., Sheldahl, L. C. & Moon, R. T. A transgenic Lef1/beta-catenin-dependent reporter is expressed in spatially restricted domains throughout zebrafish development. *Dev Biol* **241**, 229-237, doi:10.1006/dbio.2001.0515 (2002).

5 Stoick-Cooper, C. L. *et al.* Distinct Wnt signaling pathways have opposing roles in appendage regeneration. *Development* **134**, 479-489, doi:10.1242/dev.001123 (2007).

6 MacDonald, B. T., Tamai, K. & He, X. Wnt/beta-catenin signaling: components, mechanisms, and diseases. *Dev Cell* **17**, 9-26, doi:10.1016/j.devcel.2009.06.016 (2009).

7 Kagermeier-Schenk, B. *et al.* Waif1/5T4 inhibits Wnt/beta-catenin signaling and activates noncanonical Wnt pathways by modifying LRP6 subcellular localization. *Dev Cell* **21**, 1129-1143, doi:10.1016/j.devcel.2011.10.015 (2011).

8 Weidinger, G., Thorpe, C. J., Wuennenberg-Stapleton, K., Ngai, J. & Moon, R. T. The Sp1-related transcription factors sp5 and sp5-like act downstream of Wnt/beta-catenin signaling in mesoderm and neuroectoderm patterning. *Curr Biol* **15**, 489-500, doi:10.1016/j.cub.2005.01.041 (2005).

9 Yeo, S. Y., Kim, M., Kim, H. S., Huh, T. L. & Chitnis, A. B. Fluorescent protein expression driven by her4 regulatory elements reveals the spatiotemporal pattern of Notch signaling in the nervous system of zebrafish embryos. *Dev Biol* **301**, 555-567, doi:10.1016/j.ydbio.2006.10.020 (2007).

10 Filali, M., Cheng, N., Abbott, D., Leontiev, V. & Engelhardt, J. F. Wnt-3A/beta-catenin signaling induces transcription from the LEF-1 promoter. *J Biol Chem* **277**, 33398-33410, doi:10.1074/jbc.M107977200 (2002).

11 Li, T. W. *et al.* Wnt activation and alternative promoter repression of LEF1 in colon cancer. *Mol Cell Biol* **26**, 5284-5299, doi:10.1128/MCB.00105-06 (2006).

12 Mosimann, C. *et al.* Ubiquitous transgene expression and Cre-based recombination driven by the ubiquitin promoter in zebrafish. *Development* **138**, 169-177, doi:10.1242/dev.059345 (2011).

13 Peri, F. & Nusslein-Volhard, C. Live imaging of neuronal degradation by microglia reveals a role for v0-ATPase a1 in phagosomal fusion in vivo. *Cell* **133**, 916-927, doi:10.1016/j.cell.2008.04.037 (2008).

14 Knopf, F. *et al.* Dually inducible TetON systems for tissue-specific conditional gene expression in zebrafish. *Proc Natl Acad Sci U S A* **107**, 19933-19938, doi:10.1073/pnas.1007799107 (2010).

15 Matsuoka, R. L. *et al.* Radial glia regulate vascular patterning around the developing spinal cord. *Elife* **5**, doi:10.7554/eLife.20253 (2016).

16 Curado, S., Stainier, D. Y. & Anderson, R. M. Nitroreductase-mediated cell/tissue ablation in zebrafish: a spatially and temporally controlled ablation method with applications in developmental and regeneration studies. *Nat Protoc* **3**, 948-954, doi:10.1038/nprot.2008.58 (2008).

17 Bernardos, R. L. & Raymond, P. A. GFAP transgenic zebrafish. *Gene Expr Patterns* **6**, 1007-1013, doi:10.1016/j.modgep.2006.04.006 (2006).
18 Lee, R. T., Asharani, P. V. & Carney, T. J. Basal keratinocytes contribute to all strata of the adult zebrafish epidermis. *PLoS One* **9**, e84858, doi:10.1371/journal.pone.0084858 (2014).
19 Ando, K. *et al.* Clarification of mural cell coverage of vascular endothelial cells by live imaging of zebrafish. *Development* **143**, 1328-1339, doi:10.1242/dev.132654 (2016).
20 Goritz, C. *et al.* A pericyte origin of spinal cord scar tissue. *Science* **333**, 238-242, doi:10.1126/science.1203165 (2011).
21 Richardson, R. *et al.* Adult zebrafish as a model system for cutaneous wound-healing research. *J Invest Dermatol* **133**, 1655-1665, doi:10.1038/jid.2013.16 (2013).
22 Bader, H. L. *et al.* Zebrafish collagen XII is present in embryonic connective tissue sheaths (fascia) and basement membranes. *Matrix Biol* **28**, 32-43, doi:10.1016/j.matbio.2008.09.580 (2009).
23 Wang, J., Karra, R., Dickson, A. L. & Poss, K. D. Fibronectin is deposited by injury-activated epicardial cells and is necessary for zebrafish heart regeneration. *Dev Biol* **382**, 427-435, doi:10.1016/j.ydbio.2013.08.012 (2013).
24 Araya, C. *et al.* Mesoderm is required for coordinated cell movements within zebrafish neural plate in vivo. *Neural Dev* **9**, 9, doi:10.1186/1749-8104-9-9 (2014).
25 Chen, D., Jarrell, A., Guo, C., Lang, R. & Atit, R. Dermal beta-catenin activity in response to epidermal Wnt ligands is required for fibroblast proliferation and hair follicle initiation. *Development* **139**, 1522-1533, doi:10.1242/dev.076463 (2012).
26 Feng, Y., Santoriello, C., Mione, M., Hurlstone, A. & Martin, P. Live imaging of innate immune cell sensing of transformed cells in zebrafish larvae: parallels between tumor initiation and wound inflammation. *PLoS Biol* **8**, e1000562, doi:10.1371/journal.pbio.1000562 (2010).
27 Devoto, S. H., Melancon, E., Eisen, J. S. & Westerfield, M. Identification of separate slow and fast muscle precursor cells in vivo, prior to somite formation. *Development* **122**, 3371-3380 (1996).
